# Enzymatic properties, evidence for *in vivo* expression, and intracellular localization of shewasin D, the pepsin homolog from *Shewanella denitrificans*

**DOI:** 10.1038/srep23869

**Published:** 2016-03-31

**Authors:** Ana Rita Leal, Rui Cruz, Daniel Bur, Pitter F. Huesgen, Rosário Faro, Bruno Manadas, Alexander Wlodawer, Carlos Faro, Isaura Simões

**Affiliations:** 1 CNC-Center for Neuroscience and Cell Biology, University of Coimbra, 3004-517 Coimbra, Portugal; 2 Biocant, Biotechnology Innovation Center, 3060-197 Cantanhede, Portugal; 3 Actelion Pharmaceuticals Ltd, CH-4123 Allschwil, Switzerland; 4 Central Institute for Engineering, Electronics and Analytics, ZEA-3, Forschungszentrum Jülich, 52425 Jülich, Germany; 5 Protein Structure Section, Macromolecular Crystallography Laboratory, National Cancer Institute at Frederick, Frederick, MD 21702 Maryland, USA

## Abstract

The widespread presence of pepsin-like enzymes in eukaryotes together with their relevance in the control of multiple biological processes is reflected in the large number of studies published so far for this family of enzymes. By contrast, pepsin homologs from bacteria have only recently started to be characterized. The work with recombinant shewasin A from *Shewanella amazonensis* provided the first documentation of this activity in prokaryotes. Here we extend our studies to shewasin D, the pepsin homolog from *Shewanella denitrificans*, to gain further insight into this group of bacterial peptidases that likely represent ancestral versions of modern eukaryotic pepsin-like enzymes. We demonstrate that the enzymatic properties of recombinant shewasin D are strongly reminiscent of eukaryotic pepsin homologues. We determined the specificity preferences of both shewasin D and shewasin A using proteome-derived peptide libraries and observed remarkable similarities between both shewasins and eukaryotic pepsins, in particular with BACE-1, thereby confirming their phylogenetic proximity. Moreover, we provide first evidence of expression of active shewasin D in *S. denitrificans* cells, confirming its activity at acidic pH and inhibition by pepstatin. Finally, our results revealed an unprecedented localization for a family A1 member by demonstrating that native shewasin D accumulates preferentially in the cytoplasm.

Aspartic proteases (APs) of family A1 are found ubiquitously in eukaryotic organisms, whereas the presence of genes encoding these enzymes in bacteria was shown to be restricted to only seven species, five from marine bacteria and two from plant commensals^1^. However, that study was only based on analysis of gene sequences and did not provide proofs of actual expression of active APs in any bacterial species.

Based on molecular modeling it can be postulated that, similarly to their eukaryotic counterparts, pepsin homologs from marine bacteria are bi-lobal and contain two catalytic aspartates organized in the consensus sequence motif Asp-Thr/Ser-Gly, followed further downstream by the conserved hydrophobic-hydrophobic-Gly sequence, which together form the structural feature known as the psi loop. The hallmark catalytic motifs in members of family A1 are usually contained in the sequence Xaa-Xaa-Asp-Xbb-Gly-Xcc, in which Xaa is hydrophobic, Xbb is Thr or Ser, and Xcc is Ser, Thr or Ala, although the presence of an alanine residue in Xcc is much more common among APs of the retropepsin type (family A2)^2^,^3^. Interestingly, all pepsin homologs from marine bacteria display an Ala residue in this position in the second consensus motif^1^, as observed in renin (one of the exceptions in family A1). Accordingly, it was expected that these bacterial pepsin homologs would be active at weakly acidic pH, since the shift of an optimal pH to a less acidic range observed in renin and retropepsins has been partially attributed to the presence of this residue^4^. However, our results for recombinant shewasin A, the pepsin homolog from *Shewanella amazonensis*, do not support this assumption since the protease was shown to be most active at pH 4, displaying no activity at pH 6^5^, clearly suggesting some variations in its subsite binding pocket. Indeed, shewasin A displayed properties extraordinarily similar to those of pepsin-type proteases, although showing molecular features distinguishable from those of its eukaryotic counterparts, such as the lack of both signal peptide and prosegment, as well as the number of cysteine residues and their position within the sequence^5^. All in all, these observations suggest that bacterial pepsin-like APs may represent an ancestral version of modern pepsin-like enzymes, probably arising from a gene duplication and fusion event from primordial homodimeric APs. In fact, our recent identification of a gene for a single-lobed AP in *Rickettsia conorii* coding for an active enzyme with properties resembling those of retropepsins, where the monomer follows the typical fold observed in retropepsins, further corroborates this hypothesis^6^,^7^. Since the distinguishing molecular features of shewasin A are extended to the other four pepsin homologs from marine bacteria, this raises the questions of whether these proteases also share similar enzymatic properties and to what extent the properties of these bacterial pepsins are still reflected in the evolutionarily more recent members of family A1.

To start tackling these questions we expanded our investigations to shewasin D, the pepsin-like homolog from *Shewanella denitrificans* which shares 55% sequence identity with shewasin A. In this work, we describe the characterization of the recombinant protease and demonstrate that it has properties very similar to shewasin A. Moreover, we determined detailed substrate sequence specificity preferences for both shewasin D and shewasin A by a high-throughput profiling approach and clearly confirm common preferences with eukaryotic pepsin-like proteases, although subtle differences in subsite binding pockets are anticipated. Additionally, we demonstrate that shewasin D is transcribed and translated *in vivo* and provide experimental evidence that it is mainly localized in the cytosol in *S. denitrificans*.

This work provides first evidence for *in vivo* expression of a pepsin-like AP in bacteria, confirms an unprecedented cytoplasmic localization for a family A1 member, and contributes important insights to further understanding the evolutionary diversification undertaken by this important family of enzymes.

## Results

### Expression, purification, and activity of recombinant shewasin D

**Figure 1:**
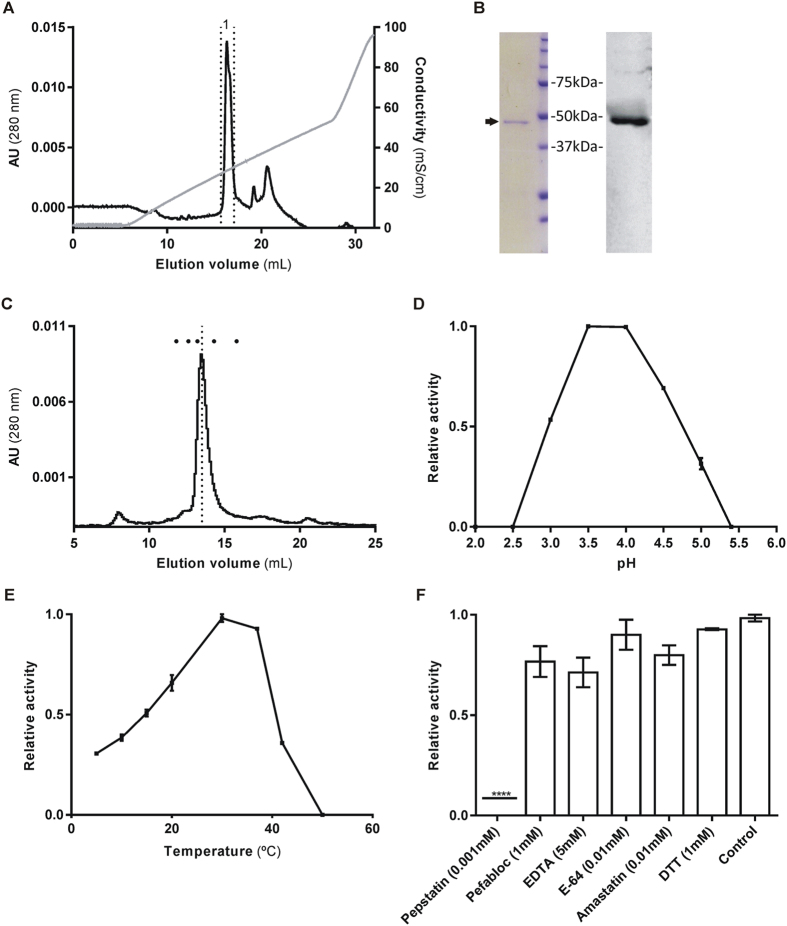
Purification and characterization of recombinant shewasin D. Wild-type shewasin D was produced in *E. coli* in a soluble form, fused to an N-terminal His-tag. A purification protocol was optimized as described in Methods. (**A**) Anion exchange chromatogram. Purified recombinant shewasin D (dotted lines, sample 1) was subsequently used in all characterization assays. (**B**) SDS-PAGE and Western blot (anti-His) analyses of sample 1, panel (**A**). (**C**) Analytical SEC of purified recombinant shewasin D. The dots indicate the elution volumes of molecular mass markers used for calibration (from left to right: aldolase, 158 kDa; conalbumin, 75 kDa; ovalbumin, 43 kDa; carbonic anhydrase, 29 kDa; ribonuclease A, 13.7 kDa). (**D**) Effect of pH on the activity of recombinant shewasin D. Shewasin D was tested for activity using the fluorogenic peptide (MCA)Lys-Lys-Pro-Ala-Glu-Phe-Phe-Ala-Leu-Lys(DNP) as substrate; activities were measured by incubating shewasin D at 37 °C with buffers between pH 2.5 and pH 5.5 containing 0.1 M NaCl. (**E**) Effect of temperature on the activity of recombinant shewasin D. Activity studies were performed by incubating shewasin D at temperatures between 5 and 50 °C, for 8 min, towards the same substrate. (**F**) Effect of protease inhibitors on the activity of recombinant shewasin D. The effect of different compounds was tested using the same fluorogenic substrate by preincubating the enzyme with each inhibitor for 8 min at 37 °C. ****P < 0.0001 compared with control using one-way ANOVA followed by Tukey’s post hoc test. The error bars represent standard deviation of the mean.

The *S. denitrificans* pepsin homolog gene shewasin D was chemically synthesized with codon usage optimized for expression in *E. coli* (Supplementary Fig. S1). The synthetic gene was fused in frame with a N-terminal His-Tag and expressed in *E. coli* C41 (DE3) strain in a soluble form. The purification protocol consisted of three chromatographic steps. First, the soluble fraction of cell lysates was applied to a His-Trap HP column loaded with cobalt. The fractions enriched in recombinant shewasin D were pooled, concentrated, and then applied to a size exclusion chromatography column. The obtained eluates were further purified by anion exchange chromatography (Fig. 1A). The effectiveness of the purification process was monitored by SDS-PAGE and Western blot analysis (Fig. 1B). The molecular mass of recombinant shewasin D was determined by analytical size exclusion chromatography under non-denaturing conditions and estimated to be ~50 kDa (Fig. 1C). This result is consistent with a monomeric state of recombinant shewasin D (theoretical: 49025 Da) and is in line with what has been previously demonstrated for shewasin A^5^.

Next, we tested the proteolytic activity of recombinant shewasin D using five different fluorogenic peptides typically used as substrates of APs with different origins. None of the three substrates: a renin substrate Arg-Glu-(EDANS)-Ile-His-Pro-Phe-His-Leu-Val-Ile-His-Thr-Lys-(DABCYL)-Arg, a BACE-1 substrate [MCA-Lys]-Ser-Glu-Val-Asn-Leu-Asp-Ala-Glu-Phe-[Lys-DNP], or [MCA-Lys]-Ala-Leu-Ile-Pro-Ser-Tyr-Lys-Trp-Ser-[Lys-DNP] was cleaved under the conditions applied. The lack of shewasin D activity towards BACE-1 substrate suggests different substrate preferences, despite the sequence similarity and relatively close phylogenetic relationship of this bacterial pepsin-like AP with mammalian BACE-1 and BACE-2^1^. Interestingly, shewasin D also failed to cleave the fluorogenic peptide (MCA)Lys-Leu-His-Pro-Glu-Val-Leu-Phe-Val-Leu-Glu-Lys(DNP), which was effectively hydrolyzed by shewasin A^5^. The only fluorogenic peptide cleaved by recombinant shewasin D was (MCA)Lys-Lys-Pro-Ala-Glu-Phe-Phe-Ala-Leu-Lys(DNP), a substrate widely used to assess proteolytic activity of A1 APs. This enzyme displayed maximum activity towards this substrate at pH values between pH 3.5 and 4.0, with a drastic decrease in activity of 50% and 70% at pH values 3.0 and 5.0, respectively (Fig. 1D). These results confirmed the optimal activity of shewasin D at acidic pH, as expected for a typical pepsin-like AP. Analysis of the cleavage products by MS revealed Phe*Phe (*indicates the cleavage site) as the major cleavage site (data not shown). The kinetic parameters for the hydrolysis of this model peptide were therefore determined at pH 4 and estimated to be *K*_m _= 8.67 μM, *k*_cat _= 3.51 s^−1^, and *k*_cat_/*K*_m _= 4.0 × 10^5 ^M^−1^. s^−1^. This *K*_m_ value was very similar to that of shewasin A^5^, but the *k*_cat_ parameter is significantly enhanced and results in a 72.3-fold higher specificity constant (*k*_cat_/*K*_m_), indicating a higher catalytic efficiency of shewasin D for this substrate. Maximal substrate turnover for (MCA)Lys-Lys-Pro-Ala-Glu-Phe-Phe-Ala-Leu-Lys(DNP) was detected between 30 °C and 37 °C. Above this temperature the activity abruptly decreased, with no detectable activity remaining at 50 °C. At lower temperatures the activity decreased gradually, with the enzyme retaining 30% activity at 10 °C (Fig. 1E).

The effect of class-specific inhibitors on the activity of purified recombinant shewasin D was also evaluated (Fig. 1F). With the exception of pepstatin A, which completely inhibited shewasin D activity (at 1 μM), no significant inhibitory effect was observed for any other tested compound. To further demonstrate the dependence of the catalytic activity on the conserved aspartate residues we generated a mutant in which the aspartate belonging to the first catalytic triad was substituted by an alanine [shewasin D_D37A]. Expression and purification were performed under identical conditions as described for wt shewasin D and analysis of the purified mutant protein by analytical size-exclusion chromatography confirmed an estimated molecular mass of ~50kDa (data not shown). However, no proteolytic activity could be detected when tested with (MCA)Lys-Lys-Pro-Ala-Glu-Phe-Phe-Ala-Leu-Lys(DNP) at pH 4.0.

### Specificity profiling with proteome-derived peptide libraries

**Figure 2:**
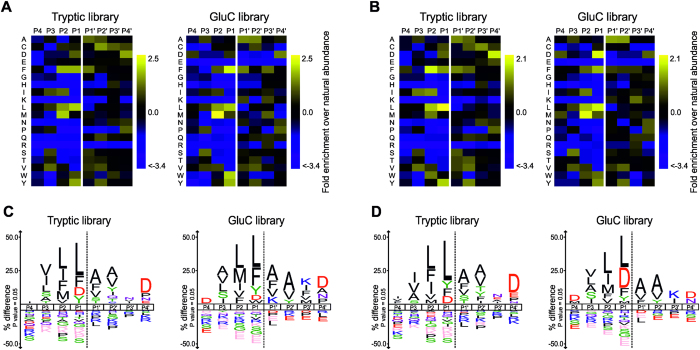
Shewasin D and shewasin A specificity preferences profiled by PICS. Graphical representation of shewasin D (**A**,**C**) and shewasin A (**B**,**D**) specificity profiles by Heatmaps and IceLogos. Results are from Tryptic and GluC peptide libraries derived from a *Homo sapiens* proteome (THP1 cells) incubated with recombinant shewasin D or shewasin A at a ratio of 1:40 (enzyme/library). The analytical strategy applied was similar to that described in ref. 9. The average amino acid occurrences in P4–P4′ were calculated from one experiment for the trypsin library and two experiments for the GluC library and are shown in the form of two-dimensional heatmaps of log(2) transformed values of fold-enrichment over natural abundance of amino acids (**A**,**B**) and % difference IceLogos (**C**,**D**). Both tryptic and GluC display consistency between them. In IceLogos representation, horizontal axis represents the amino acid position and vertical axis denotes the over- and under-representation of amino acid occurrence compared with the Swiss-Prot Homo sapiens protein database. Cysteines are carboxyamidomethylated and lysines are dimethylated.

The activity assays that utilized the synthetic fluorogenic substrates suggested some differences in specificity preferences between shewasin D and shewasin A. To obtain a more general view, we determined the sequence specificity profile for both recombinant shewasins using Proteomic Identification of protease Cleavage Sites (PICS)^8^,^9^. This approach is based on the identification of hundreds of cleaved peptide sequences after incubation with database-searchable, proteome-derived peptide libraries, which enables the determination of both prime and nonprime specificity preferences of the protease of interest in a single experiment. Two complementary peptide libraries prepared by digestion of human THP1 cell protein extracts with trypsin or GluC, respectively, were independently incubated with purified recombinant shewasin D or shewasin A. Selective enrichment and mass spectrometric analysis identified 1341 C-terminal cleavage products from the tryptic library and 322 C-terminal cleavage products from the GluC library for shewasin D, as well as 1262 and 393 C-terminal cleavage products from tryptic and GluC libraries, respectively, for shewasin A. Database searches using the WebPICS tool^9^ inferred the corresponding non-prime side products and hence the complete cleavage site sequences. Alignment of the cleavage sites and normalization to the natural abundance of each amino acid in the human proteome revealed the specificity profiles of recombinant shewasin D and shewasin A (Fig. 2). Consistent results were obtained for each protease using the two complementary peptide libraries. Interestingly, the PICS specificity profiles of shewasin D and A were very similar. Both enzymes accommodated a variety of mostly hydrophobic residues in their substrate binding clefts, with a slightly stronger restriction on the non-prime side. Positively ionizable amino acids such as histidine, lysine, and arginine, as well as the neutral asparagine and glutamine were underrepresented at positions ranging from P1 to P4^10^. At P1, directly preceding the cleaved peptide bond, both proteases strongly preferred leucine and aromatic amino acids, such as phenylalanine and tyrosine. Interestingly, aspartate was also clearly overrepresented; however, at the applied pH this amino acid is likely to be protonated to a significant degree and therefore uncharged. Proline was never found in this position, while branched aliphatic amino acids, the neutral amino acids serine, threonine, glutamine, asparagine, and positively charged amino acids and the achiral glycine were all underrepresented. On the C-terminal side of the scissile bond in P1′, both enzymes preferred neutral alanine, phenylalanine, valine, and serine. Although other hydrophobic amino acids could be accommodated at this position, leucine was underrepresented together with proline, histidine, and arginine. At P2, a strong preference for hydrophobic amino acids (leucine, phenylalanine, isoleucine, valine, and methionine) was observed, while charged and neutral amino acids are clearly disfavored, as already found in P1. This trend for the preferential accommodation of hydrophobic amino acids and exclusion of charged amino acids was maintained in P2′, as well as in positions more distant from the cleavage site, such as P3. Interestingly, position P3′ appeared to be much less stringent, accepting all amino acids except proline. Notably, a clear preference for aspartate and proline was observed in P4′, whereas charged and neutral amino acids were underrepresented in P4.

Given the similar profiles observed for both proteases, the highly different activity towards the peptide (MCA)Lys-Leu-His-Pro-Glu-Val-Leu-Phe-Val-Leu-Glu-Lys(DNP), which was cleaved by shewasin A at Leu*Phe but not by shewasin D, was striking.

**Figure 3:**
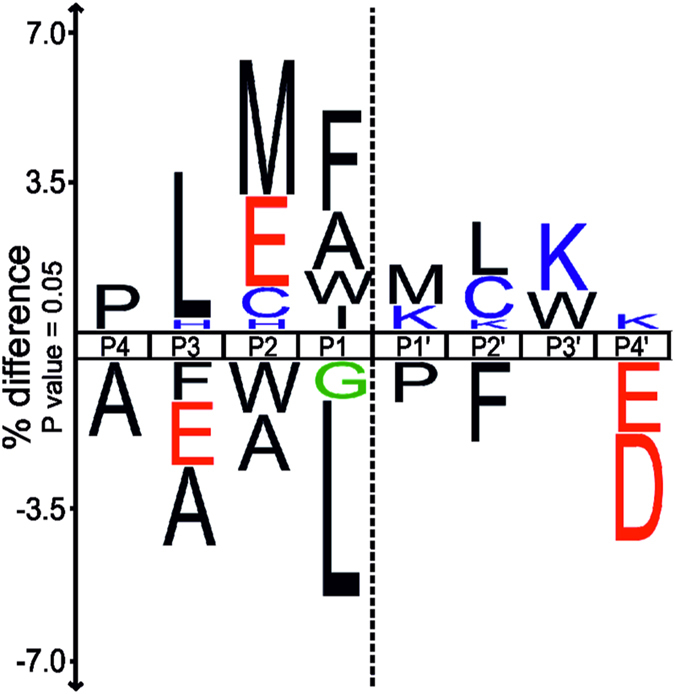
Differential IceLogo representation of shewasin D versus shewasin A specificity profile using trypsin PICS library. The differential IceLogo represents the enriched and depleted residues for shewasin D (upper part of the IceLogo) using as reference set the peptides identified for shewasin A. Residues that are over- or under-represented in the experimental set are respectively shown in the top or bottom part of the IceLogo.

To investigate whether PICS data could reveal an indication for the underlying reason, we further analyzed the tryptic datasets, where large numbers of cleaved sites were identified for both enzymes. First, we generated a differential sequence logo of the tryptic datasets, illustrating residues enriched or depleted at each position in shewasin D as compared to shewasin A (Fig. 3). As expected, the differences were rather small. Shewasin D showed a stronger preference for aromatic amino acids including phenylalanine at P1, whereas shewasin A cleaved more peptides with leucine at this position. In addition, shewasin D showed a preference for methionine and glutamate at P2 as well as for leucine at P3. At P4′, peptides cleaved by shewasin D were slightly depleted in negatively charged amino acids when compared with shewasin A. Second, we explored subsite cooperativity between P3-P3′ positions^11^. No strong differences in subsite preference were observed when only the cleaved sequences with Leu at P1 were considered (data not shown). Fewer cleaved sequences with phenylalanine at P1′ contained leucine at P1 as compared to the whole dataset, but this effect was similar for both shewasin D and shewasin A (Supplementary Fig. S2A,B). Peptides with a branched valine at P2′ disfavored leucine at P1 slightly more for shewasin D than A (Supplementary Fig. S2C,D), but again the difference in selectivity was rather low. These results suggest that the selectivity of shewasins may likely extend beyond the P3–P3′ positions.

Subsite cooperativity was also explored to provide additional explanation to the observed differences in the catalytic efficiency between shewasins. A phenylalanine in P1 disfavored the presence of a bulky phenylalanine in P1′ in the shewasin A dataset (Supplementary Fig. S2E,F). Moreover, the simultaneous accommodation of phenylalanine residues by subsites S1 and S1′ may be facilitated by the presence of the glutamate residue in the P2 position of the substrate. For both shewasins, glutamate is more frequently found at P2 if P1′ is occupied by phenylalanine (Supplementary Fig. S2A,B) and vice versa Supplementary Fig. S2G,H). Taken together, these individual small differences in the active site structure may add up and produce the higher efficiency of shewasin D cleavage of the peptide (MCA)Lys-Lys-Pro-Ala-Glu-Phe-Phe-Ala-Leu-Lys(DNP) at the Phe*Phe bond.

### Native shewasin D is active and inhibited by pepstatin

**Figure 4 f4:**
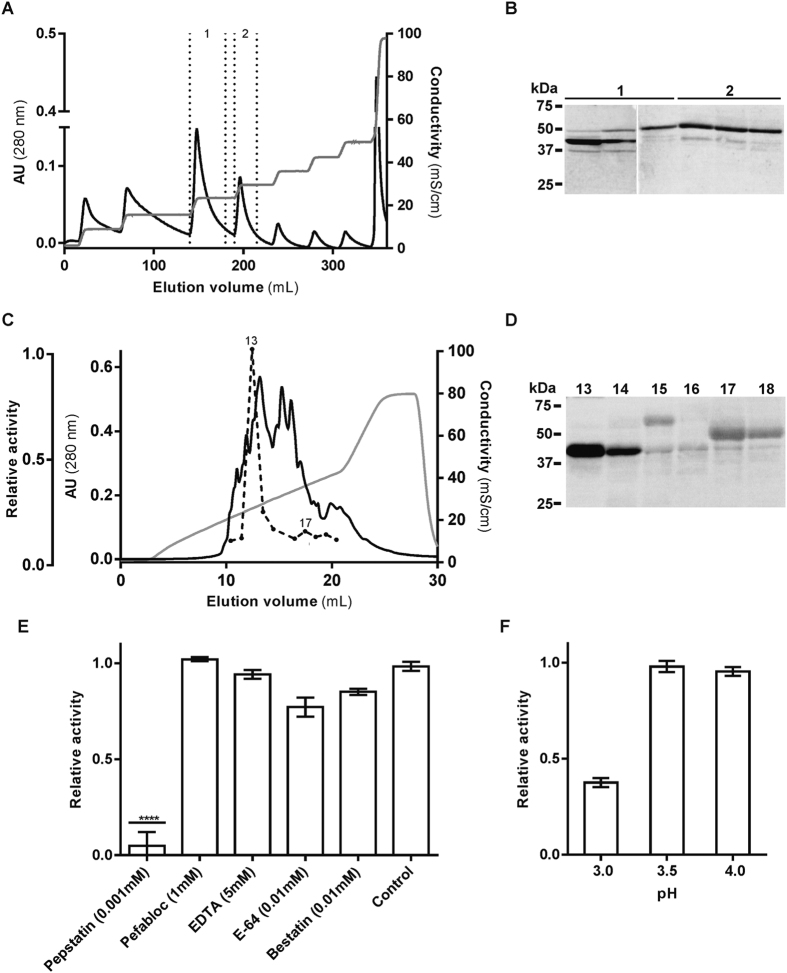
Partial purification of native shewasin D. A soluble protein extract of *S. denitrificans* overnight cultures was prepared and subjected to two anion exchange chromatographic steps. (**A**) High Q chromatogram of soluble extract fractionation. Dotted lines delimit the fractions staining positive in the Western blot analysis with anti-shewasin D antibody. (**B**) Western blot analysis of samples 1 and 2 (panel **A**), with the anti-shewasin D antibody. (**C**) Mono Q chromatogram. Fractions 1 and 2 from (**A**) were pooled, diluted and subsequently applied to a Mono Q column. The enzymatic activity was tested towards the substrate (MCA)Lys-Lys-Pro-Ala-Glu-Phe-Phe-Ala-Leu-Lys(DNP) and is represented by a discontinuous line. (**D**) Western blot analysis of fractions 13–18 eluted from Mono Q (**C**) with anti-shewasin antibody. (**E**) Effect of protease inhibitors on the activity of partially purified fractions of native shewasin D. The effect of different compounds was tested by preincubating protein sample (fraction 13, panel **C**) with each inhibitor for 8 min at 37 °C. ****P < 0.0001 compared with control using one-way ANOVA followed by Tukey’s post hoc test. (**F**) Effect of pH on the activity of partially purified fractions of native shewasin D. The assays were performed by incubating fraction 13 at 37 °C with buffers between pH 3.0 and 4.0. The error bars represent standard deviation of the mean.

We have shown clear evidence that the *S. denitrificans* pepsin homolog gene encodes for an active enzyme (with typical AP properties), thus corroborating previous work with its *S. amazonensis* ortholog^5^ and confirming that both bacterial gene sequences encode functional enzymes. However, the fact that the recombinant enzyme is active does not by itself prove its presence and activity in the original bacterial species. To further investigate whether shewasin D is expressed *in vivo*, we first confirmed the presence of transcripts by RT-PCR analysis (Supplementary Fig. S3) as well as protein expression in total extracts of *S. denitrificans* cells by Western blot analysis, which detected two bands migrating at an apparent MW of approximately 40 kDa and 50 kDa (Supplementary Fig. S4). We then attempted to purify native shewasin D by pepstatin-agarose affinity chromatography and again detected two bands with our shewasin D antibody (Supplementary Fig. S5). However, given the low yield of this purification method it was impossible to further separate the two forms. Next we implemented a different partial purification approach. A soluble protein extract prepared from overnight cultures was applied to an ion-exchange Mini Macro-Prep High Q Cartridge column (5 mL) (Bio Rad) and the eluted fractions were analyzed by Western blot with shewasin D specific antibody (Fig. 4A[Fig f4]B). The two reactive species were detected in the fractions eluted with a salt concentration between 190 and 250 mM. Given the theoretical molecular weight of shewasin D (~46.7 kDa), these results suggested that either native shewasin D undergoes some proteolytic cleavage or that the lower molecular weight band may result from protein degradation. To further evaluate this finding these fractions were pooled and subjected to a second anion-exchange chromatography on a Mono Q column. The presence of shewasin D in eluted fractions was again confirmed through Western blot and followed by enzymatic activity assays at pH 4.0 with the fluorogenic peptide (MCA)Lys-Lys-Pro-Ala-Glu-Phe-Phe-Ala-Leu-Lys(DNP), shown to be cleaved by the recombinant enzyme. As illustrated in Fig. 4C,D, the eluted fraction displaying the highest activity towards this substrate showed a reactive band of ~ 40 kDa in the Western blotting, whereas lower enzymatic activity was observed in the fraction enriched in the 50 kDa species. These results appear to suggest that native shewasin D may likely occur in a processed as well as in a precursor (or autoinhibited form). To further confirm the nature of the observed enzymatic activity, this partially purified fraction enriched in the 40 kDa band was subsequently tested for activity in the presence of protease inhibitors. As expected, only pepstatin A had an inhibitory effect on this proteolytic activity (Fig. 4E). Moreover, the activity was assayed at pH 3.0, 3.5, and 4.0, and consistent with the results obtained with the recombinant enzyme, fractions enriched in native shewasin D showed similar enzymatic activity when tested at pH 3.5 and pH 4.0, with a comparable decrease in activity at pH 3.0 (Fig. 4F).

### Intracellular localization of native shewasin D

**Figure 5:**
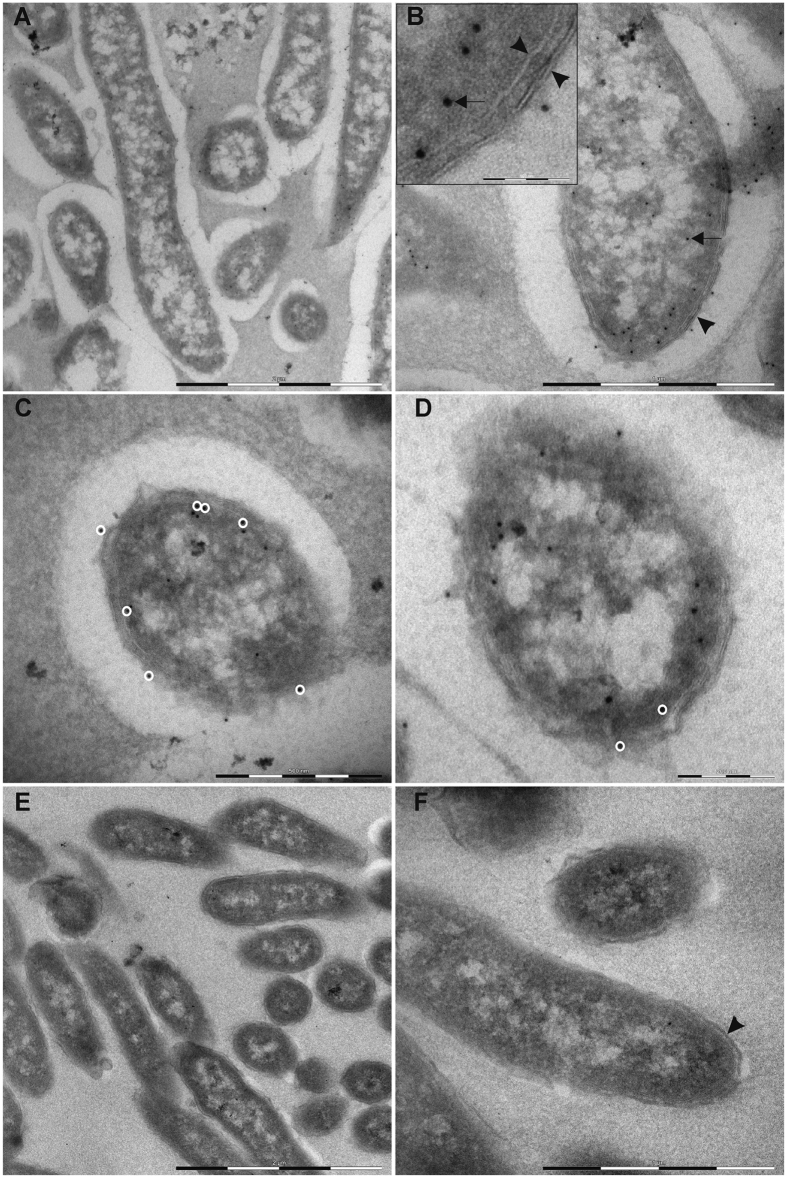
Shewasin D is preferentially accumulated in the cytoplasm in *Shewanella denitrificans*. Representative iEM images of *Shewanella denitrificans* immunogold labeled using anti-shewasin D (1:200) as primary antibody (**A–D**) or pre-immune serum (1:16 000) (**E**,**F**). Both membranes (arrowheads), the periplasm between them as well as the gold particles (arrows) can be discerned (**B**). Gold particles within 25 nm from the membrane bilayers are encircled (**C**,**D**). The white spaces surrounding the cells correspond to bacterial capsules. Scale bar represents 2 μm (**A**,**E**), 1 μm (**B**,**F**), 100 nm (**B**, inset), 500 nm (**C**) and 200 nm (**D**).

Next we determined the localization of shewasin D in *S. denitrificans* cells by immunogold labeling. The acquired immuno-electron microscopy (iEM) images of *S. denitrificans* bacteria showed sufficient morphological preservation to discern the double membrane bilayers (Fig. 5B, inset) and some intracellular structures. A distinct difference in the number of gold particles per bacterium in the anti-shewasin D labeled sample (Fig. 5A,B) compared to the pre-immune serum control (Fig. 5E,F) was evident. The colloidal gold particles, 10 nm in diameter, were conjugated to Staphylococcal Protein A (sPA), with a length of approximately 5 nm. Since the primary antibodies are about 15 nm in length, the center of the colloidal gold particle can be localized up to 25 nm from the antigen to which it is interconnected. Although the gold particles appeared to preferentially accumulate in the vicinity of the lipid bilayers in the anti-shewasin D labeled samples, only a fraction of the gold particles were within a 25 nm radius from either the inner or outer membrane (Fig. 5C,D). These results suggest that the antigen accumulates in the cytoplasm, with only a subfraction of the antigen being localized to the membranes or the periplasm. Interestingly, the majority of gold particles overlaid the electron dense cytoplasmic areas, while localization to the lightly stained intracellular regions could seldom be observed. Only rarely could gold particles be found in the extracellular space, indicating that the antigen is not secreted.

## Discussion

Pepsin homologs from bacteria have been identified through bioinformatics analysis^1^ but their enzymatic properties, native expression, intracellular localization, as well as structure-function relationships are still far from having been elucidated. Thus far, an enzymatic characterization has been provided for only shewasin A from *S. amazonensis*^5^. To gain further insights into this group of bacterial members of family A1 proteases we expanded our studies to the homolog from Gram-negative *S. denitrificans*, thereby named shewasin D. Shewasins A and D share 55% of amino acid sequence identity and are phylogenetically close to the eukaryotic aspartic proteases BACE-1 and BACE-2^1^. Here we report the characterization of recombinant shewasin D and the first comprehensive cleavage specificity profiles of both shewasin D and shewasin A. Moreover, our data provide the first experimental evidence that shewasin D is expressed *in vivo* in *S. denitrificans* cells and demonstrate that it accumulates preferentially in the cytoplasm.

In general, enzymatic properties of recombinant shewasin D were shown to be very similar to those previously described for shewasin A and other pepsin-like APs. Like shewasin A, shewasin D was shown to be monomeric, to be specifically inhibited by pepstatin, to depend on an intact Asp-Asp catalytic dyad for proteolytic activity, and to display maximum activity at acidic pH towards a substrate typically used to assess activity of APs, also hydrolyzing it between two hydrophobic residues. The optimal pH of shewasin D between pH 3.5 and 4.0 was slightly lower if compared to shewasin A, which is most active between pH 3.75–4.5^5^. The temperature profile of recombinant shewasin D was shifted towards lower temperatures, with maximal activity detected at 30 °C to 37 °C, compared to 42 °C to 50 °C for shewasin A^5^, likely reflecting physiological characteristics intrinsic to each *Shewanella* species^12^,^13^. However, the remarkable difference in activity towards (MCA)Lys-Lys-Pro-Ala-Glu-Phe-Phe-Ala-Leu-Lys(DNP) with specificity constants of 4.0 × 10^5 ^M^−1^. s^−1^ and 5.6 × 10^3 ^M^−1^. s^−1^ 5 for shewasin D and A, respectively, and the distinct preferences for the synthetic substrate (MCA)Lys-Leu-His-Pro-Glu-Val-Leu-Phe-Val-Leu-Glu-Lys(DNP), which was not cleaved by shewasin D, clearly point to differences in either the architecture or properties of their substrate-binding pockets.

To further explore specificity preferences of both proteases and compare them with their eukaryotic counterparts we determined P4–P4′ specificity profiles using PICS^8^. This type of proteomics analysis has been reported for HIV-1 protease^9^ and recently for the retropepsin-like APRc from *Rickettsia*^6^; however, to our knowledge, this is the first report on the use of this experimental approach for the characterization of pepsin-type AP specificity, as for these enzymes specificity studies have been essentially performed with individual substrates or using combinatorial peptide libraries^14^,^15^,^16^,^17^,^18^,^19^,^20^. Our data clearly confirmed common sequence preferences for both shewasins and eukaryotic pepsins. These include a strong preference for large hydrophobic amino acids such as leucine, phenylalanine, and tyrosine at P1 that is also described for pepsin, plasmepsins, cathepsins D and E, BACE1, cardosin A, and SAPs^15^,^16^,^17^,^18^,^19^,^20^. Also in agreement with what has been reported for most of these proteases^14^,^15^,^16^,^17^,^19^,^20^, the two β-branched hydrophobic amino acids isoleucine and valine as well as positively charged amino acids, neutral amino acids and proline were highly disfavored at this position for both shewasins. Interestingly, a strong preference for aspartate at P1 position was also noted in this study; however, aspartate might be protonated to a significant degree and therefore neutral at the applied pH. The acceptance of aspartate at the S1 pocket was already observed for porcine pepsin^14^ as well as Sap 3^19^, but with very low cleavage frequencies. A comparison of the putative amino acid residues in the S1 subsite in shewasins with those identified for porcine pepsin and BACE-1^21^,^22^ indicate some differences in the hydrophobicity of this subsite that might account for the higher preference of aspartate at P1 in shewasins. In line with what has been generally observed for pepsin-type proteases, the S1 subsite is one of the most discriminating ones, and also much more stringent than S1′ subsite. Additionally, the trend to accept preferentially hydrophobic amino acids in P1′ is obvious in both shewasins^16^,^17^,^19^,^20^. However, we observed that bacterial proteases also accept amino acids with neutral side chains, such as asparagine, glutamine, and serine at this position. Regarding the observed preferences in positions distal to the scissile bond, shewasins’ S2 subsite was unveiled to be quite stringent with similar selectivity as the S1 subsite, except for isoleucine and valine which were shown to be accommodated in S2. The conserved preference for hydrophobic amino acids in P3 and for alanine and valine at P2′ positions are also common to pepsin, plasmepsins, and BACE-1^16^,^18^,^20^. Interestingly, the pronounced lack of stringency in positions P3′ (and P4′) resembles what has been described for BACE-1^20^, including the observation that P positions are in general more specific than P′ positions^20^. However, the marked exclusion of asparagine from position P2 observed for both shewasins might explain the lack of activity towards BACE-1 substrate, since asparagine is one of the amino acids preferentially accommodated at this position by BACE-1^20^. Although PICS is an end-point assay performed at high enzyme-to-substrate ratios and thus aggregates both kinetically preferred and very modest substrates in one profile, our observations that P1′ phenylalanine favors glutamate in P2 is in agreement with results obtained for plasmepsins when using a combinatorial library pool of fixed P1′-Phe^16^, as well as for cathepsin D and pepsin^18^. Taken all together, our PICS analysis provides clear indications that both shewasins share common traits with eukaryotic pepsin-type proteases and further confirms the relevance of this experimental approach to help understanding both primary and secondary specificity determinants of these proteases. Nevertheless, our results also demonstrate that both shewasins appear to be more stringent than most eukaryotic APs, sharing more similarities with BACE-1, as anticipated by the phylogenetic analysis. Determination of the three-dimensional structure of shewasin D or shewasin A will be essential for better understanding of the observed subtle differences and the influence of interacting amino acid residues in each subsite, not only for defining specificity but also their unexpected optimum acidic pH.

Indeed, our partial purification of native shewasin D confirmed activity at acidic pH values and inhibition by pepstatin, providing the evidence that the native enzyme appears to assume the behavior of a typical pepsin-like protease *in vivo*, as shown for the recombinant form. The observed higher enzymatic activity of an apparently proteolytically processed form of native shewasin D further suggests that there might be additional regulatory mechanisms to control enzyme activity, likely compensating for the lack of the typical prosegment domain. Although further studies are required to understand the regulation of shewasin D activity and its function *in vivo*, the evidence reported herein on its predominant cytosolic localization is consistent with the lack of a signal peptide. Regulated proteolysis in bacteria has been gaining relevance in recent years as an important control mechanism of physiological processes and stress responses^23^. So far, bacterial proteases have been implicated in the regulation of specific processes, such as cell division or methionine synthesis, as well as in global responses like protein quality control, SOS, heat or envelope stress responses^23^. However, it is likely that many proteolysis-dependent processes remain to be identified^23^, especially considering that some families of proteases have only recently been identified in Gram-negative bacteria, as is the case of aspartic proteases of both A1 and A2 families^5^,^6^,^24^. Proteolytic events are generally associated with fast responses in bacteria and, therefore, must be tightly regulated. Our results showing the presence of two forms of shewasin D suggest that this protease may occur in a latent form in the cytoplasm that may be activated only under temporary local acidic conditions. Although we have been able to produce full-length shewasin D in *E. coli* in an active form, we cannot exclude the possibility that different regulatory mechanisms may indeed take place in *S. denitrificans* to maintain shewasin D in a latent form, as no homolog proteases are present in *E. coli*. Regulation of cytoplasmic pH in Gram-negative bacteria has been shown to be essential for their growth and survival, and acidification has been reported as a response to electrophilic stress^25^,^26^, hence shewasin D activation and activity may be tightly coupled with these changes in pH homeostasis and contribute to stress tolerance. Alternatively, we cannot rule out the secretion of shewasin D through a signal peptide-independent route, as suggested for other bacterial extracellular proteases^27^, that could also justify activation through processing upon secretion. The bacterial extracellular proteases reported so far play functions related to toxicity, virulence, or to nutrient acquisition and proliferation^27^,^28^,^29^,^30^, but again no detailed functional studies are yet available for bacterial pepsin-like proteases. The conservation in these genes together with their unusual pH optimum, indicates a unique kind of task these proteases have to fulfill and that is clearly worth being further uncovered.

## Materials and Methods

### Cloning of *S. denitrificans* gene encoding for shewasin D

The sequence encoding the pepsin homologue gene of *S. denitrificans* (gene locus Sden_0804; sequence available at the EBI Data Bank under the accession number ABE54094.1) was optimized for codon usage in *E. coli*. The optimized gene was chemically synthesized (Genscript, Piscataway, NJ) with restriction sites for NdeI at the 5′-end and XhoI at the 3′-end, for subcloning in pET28a (Novagen, Gibbstown, USA) in frame with a N-terminal His-Tag. The active site mutant of shewasin D (D37A) was generated using the QuikChange Site-Directed Mutagenesis Kit (Stratagene, La Jolla, USA) with the primers 5′ AAA GTG AAC CTG ATT ATT G**CG** ACC GGC AGC AGC ACC CTG 3′ (forward) and 5′CAG GGT GCT GCT GCC GGT **CG**C AAT AAT CAG GTT CAC TTT 3′ (reverse) (mutation represented in bold). Both constructs were confirmed by DNA sequencing.

### Expression and purification of recombinant shewasin D and active site mutant in *E. coli*

Wild-type shewasin D and active site mutant [shewasin D_D37A] constructs were transformed into *E. coli* C41(DE3) strain (Lucigen, WI, USA). In both cases gene expression was induced by 0.05 mM IPTG when the cultures reached an OD_600nm_ of 0.6–0.7, and protein expression was carried out overnight at 18 °C. After expression, cells were harvested by centrifugation at 8983 × *g* at 4 °C for 20 min and resuspended in 20 mM sodium phosphate buffer pH 7.5 with 10 mM imidazole and 0.5 M NaCl (binding buffer for IMAC). Lysozyme was added to a final concentration of 0.05 mg/mL and the final suspension was frozen. The same purification procedure was used for both wild type (wt) shewasin D and active-site mutant. Each suspension was thawed and DNase and MgCl_2_ were added to the final concentration of 0.05 mg/mL and 10 mM, respectively, and the reaction mixture was incubated for 2 h at 4 °C. Soluble and insoluble fractions were separated by centrifugation of the cell lysate at 12 000 × *g* for 20 min. The soluble fraction obtained was filtered through 0.2 μm filters and immediately loaded on a HisTrapHP 5 mL column (GE Healthcare Life Sciences, Uppsala, Sweden), charged with Co^2+^ ions (which was shown to be more efficient in eliminating lower molecular weight contaminants than an IMAC column loaded with Ni^2+^) and equilibrated in 20 mM sodium phosphate buffer pH 7.5 with 10 mM imidazole and 0.5 M NaCl (binding buffer). After sample loading, the column was connected to a chromatographic system (DuoFlow-BioRad, Hercules, USA) and extensively washed with binding buffer until the A_280nm_ reached a steady baseline. Protein elution was performed with a stepwise gradient of imidazole concentration (50 mM, 100 mM and 500 mM), in the same buffer. Both proteins were eluted with buffer containing 100 mM imidazole. Fractions were pooled, concentrated (Amicon Ultra Centrifugal Filter Unit, Millipore) and applied to a size exclusion chromatography HiLoad 26/60 Superdex 200 prep grade column (GE Healthcare Life Sciences), pre-equilibrated in 20 mM Tris-HCl pH 8.0 buffer with 150 mM NaCl. Shewasin D and active site mutant containing fractions were further purified by ion exchange chromatography in a Mono Q 5/50 (GE Healthcare Life Sciences) connected to an FPLC system (DuoFlow-BioRad, Hercules, USA). Protein elution was carried out with a linear gradient of NaCl (0–1 M) in 20 mM Tris-HCl pH 8.0 buffer.

### Size-exclusion chromatography

The molecular masses of purified active recombinant shewasin D and shewasin D_D37A were evaluated by size-exclusion chromatography under non-denaturing conditions on a Superose 12 (GE Healthcare Life Sciences) column equilibrated with 20 mM Tris-HCl buffer pH 8.0 with 150 mM NaCl in a DuoFlow-BioRad FPLC system. The column was calibrated with Gel Filtration HMW and LMW calibration kits (GE Healthcare Life Sciences), using as molecular mass markers aldolase (158 kDa), conalbumin (75 kDa), ovalbumin (43 kDa), carbonic anhydrase (29 kDa), and ribonuclease A (13.7 kDa).

### Enzyme assays

The proteolytic activity of purified recombinant shewasin D was determined with several fluorogenic protease substrates: [(7-methoxycoumarin-4-yl)acetyl(MCA)-Lys]-Lys-Pro-Ala-Glu-Phe-Phe-Ala-Leu-[Lys-2,4-Dinitrophenyl (DNP)] (GenScript, Piscataway, USA), [MCA-Lys]-Leu-His-Pro-Glu-Val-Leu-Phe-Val-Leu-Glu-[Lys-DNP] (Genosphere Biotechnologies), [MCA-Lys]-Ala-Leu-Ile-Pro-Ser-Tyr-Lys-Trp-Ser-[Lys-DNP] (GenScript, Piscataway, USA), the renin substrate Arg-Glu(5-[(2-aminoethyl)amino]naphthalene - 1 - sulfonic acid (EDANS)-Ile-His-Pro-Phe-His-Leu-Val-Ile-His-Thr-Lys(4- (dimethylaminoazo) benzene-4-carboxylic acid (DABCYL)-Arg (Sigma) and BACE-1 substrate [MCA-Lys]-Ser-Glu-Val-Asn-Leu-Asp-Ala-Glu-Phe-[Lys-DNP] (Genosphere Biotechnologies). Excitation and emission wavelengths of 328 nm and 393 nm, respectively, were used to monitor the rate of hydrolysis of the substrates, except for the renin substrate where an excitation wavelength of 340 nm and emission wavelength of 490 nm was used. The total fluorescence change that occurred upon complete hydrolysis of the substrate was used to determine the relationship between fluorescence change and substrate concentration. Of all the substrates listed above, only [(MCA)-Lys]-Lys-Pro-Ala-Glu-Phe-Phe-Ala-Leu-[Lys(DNP)] was found to be cleaved by recombinant shewasin D. Kinetic parameters for the cleavage of this substrate were calculated from the Lineweaver–Burk plot with appropriate software. For activity studies at different pH values (at 37 °C), the following buffers with pH values between 2.5 and 5.5 were used: 0.05 M sodium citrate (pH 2.5–3.5) and 0.05 M sodium acetate (pH 4–5.5), all containing 0.1 M NaCl. To assess activity of recombinant shewasin D at different temperatures, the proteolytic activity was determined after pre-incubation of the enzyme in 0.05 M sodium acetate buffer pH 4.0 with 0.1 M NaCl at temperatures between 5 °C and 50 °C, for 8 min. The effect of various protease inhibitors on proteolytic activity was evaluated by pre-incubating the enzyme with each compound at 37 °C, in 0.05 M sodium acetate buffer pH 4.0 with 0.1 M NaCl for 8 min, before determination of proteolytic activity. The rate of hydrolysis of the substrate was monitored for 600 seconds. Statistical analysis was performed by a one-way analysis of variance (ANOVA) followed by post hoc multiple comparisons using Tukey’s test. A *P*-value < 0.05 was taken as statistically significant. Shewasin D_D37A was tested for activity under the same assay conditions. Partially purified samples containing native shewasin D (see below) were assayed for activity towards [(MCA)-Lys]-Lys-Pro-Ala-Glu-Phe-Phe-Ala-Leu-[Lys(DNP)] at 37 °C, in 0.05 M sodium acetate buffer (pH 3–4) with 0.1 M NaCl. The effect of protease inhibitors was assayed under similar conditions as those described for recombinant shewasin D.

### RT-PCR analysis

*S. denitrificans* strain was obtained from the collection of the Centre de Ressources Biologiques de l′Institut Pasteur (CRBIP) (code 107825), and propagated under the following conditions: Difco Marine Broth (BD) (55.1 g/L) pH 7.6, agar (when needed) (15 g/L) at 30 °C. Total RNA was isolated from 1 mL of *S. denitrificans* culture using TRIzol (Invitrogen). The quality and integrity of the extracted total RNA was evaluated on 1% agarose gel electrophoresis. Total RNA sample was then treated with DNase (RNase Free, New England BioLabs) before cDNA synthesis. 1 μg of DNA-free RNA was used as template in the synthesis of single-stranded cDNA with the 1st Strand cDNA Synthesis Kit for RT-PCR (AMV) (Roche Applied Science) using the specific primer for shewasin D 3′-end: 5′ TCA TGA TTT TTG CCT ATG GGC 3′. In parallel, a negative control was performed where the reverse transcriptase was excluded from the reaction mixture. The resulting products of the single-stranded cDNA synthesis and negative control were then used as templates in PCR amplification using HotMaster Taq DNA Polymerase (5Prime) and the specific primers for *S. denitrificans* shewasin D gene (5′ CTA CCC AGC GCC CTT GAG G 3′ as the forward primer and again 5′ TCA TGA TTT TTG CCT ATG GGC 3′ as the reverse primer). The amplification conditions were as follows: 95 °C for 5 min followed by 30 cycles of 95 °C for 35 s, 58 °C for 50 s and 68 °C for 1 min. A negative control of the PCR amplification was performed where no cDNA template was added. The results of the RT-PCR were evaluated on a 1% agarose electrophoresis gel stained with ethidium bromide. The amplified product, that should correspond to the last 486 bp of the coding sequence of shewasin D, was purified (NZYGelpure, NZYtech), cloned, and both strands were sequenced by automated DNA sequencing.

### Partial purification of native shewasin D

A *S. denitrificans* culture was grown in a final volume of 2 L of Marine broth pH 7.6 at 30 °C for 16 h. The culture was then harvested by centrifugation, resuspended in 0.02 M Tris-HCl buffer pH 8.0 and the cell suspension lysed with three passages through an EmulsiFlex (AVESTIN, Inc.) (10000 psi). The soluble fraction separated by centrifugation (186000 × *g*, for 20 min) was then applied to a High Q column (Bio-Scale Mini, UNOsphere Q Cartridge, 5 mL, BioRad) connected to an FPLC system (DuoFlow-BioRad, Hercules, USA). Protein elution was carried out with a linear gradient of NaCl (0–1 M) in 0.02 M Tris-HCl buffer pH 8.0 buffer at 2 mL/min. Fractions that were immunostained with the anti-shewasin D antibody (GenScript, Piscataway, USA) were pooled, diluted and applied to a Mono Q 5/50 (GE Healthcare Life Sciences) connected to an FPLC system (DuoFlow-BioRad, Hercules, USA). Protein elution was carried out with a linear gradient of NaCl (0–1 M) in 20 mM Tris-HCl buffer pH 8.0.

### Immunogold labeling

Determination of the intracellular localization of shewasin D in *S. denitrificans* was performed by Vironova AB (Stockholm, Sweden) using the Tokuyasu method. The formaldehyde fixed (3% formaldehyde in a 0.1 M phosphate buffer, pH 7.4) *S. denitrificans* bacteria were embedded in gelatine, infiltrated with sucrose for cryoprotection, and frozen by immersion in liquid nitrogen. Ultrathin sections were prepared using a cryo-ultramicrotome under cryo conditions (–120 °C) and sections were transferred to nickel/formvar grids. Unspecific antibody binding was blocked by incubating the sections twice in 0.1 M cacodylate buffer containing 2% bovine serum albumin (BSA) and 2% gelatine. The samples were subsequently immunolabeled overnight with an anti-shewasin D antibody (diluted 1:200 to a final protein concentration of 2.5 μg/ml) or pre-immune serum (diluted 1:16000 to a final protein concentration of 2.7 μg/ml) diluted in 0.1 M cacodylate buffer containing 0.5% BSA and 0.2% gelatin. Staphylococcal Protein A (sPA) conjugated with 10 nm colloidal gold particles was used as the secondary probe. The samples were incubated with sPA-Au diluted 1:100 for 2 h and post-fixed with 2% glutaraldehyde. Finally, specimen contrast was enhanced using 0.05% uranyl acetate stabilized with 1% methylcellulose and the samples were analyzed using a FEI Tecnai 12 Biotwin transmission electron microscope. Images were acquired using a 2kx2k Veleta CCD camera (Olympus Soft Imaging Solution). Distance measurements were carried out using the Vironova Analyzer^®^ software.

### Proteomic identification of protease cleavage sites (PICS)

Recombinant shewasin D and shewasin A^5^ specificity profiling was determined by PICS methodology as described in reference^9^, with minor changes. Proteome-derived peptide libraries were generated by digest of THP-1 cells lysates with trypsin or GluC. Both libraries were independently incubated with purified recombinant shewasin D or shewasin A in 50 mM sodium acetate buffer pH 4.0 supplemented with 100 mM NaCl at protease-to-library ratios of 1:40 (w/w), for six h at 37 °C. Reactions were stopped by addition of 1 μM pepstatin. Carboxy-terminal peptide cleavage products enriched by biotinylation and streptavidin-mediated pullout, were separated on a HALO^TM^ C18 column (Eksigent) connected to a TripleTOF 5600 (AB SCIEX). Peptide sequences were identified with X!Tandem^31^, in conjunction with IProphet^32^ at a confidence level >99%. Search parameters included mass tolerances of 0.05 Da for parent ions and 0.1 Da for fragment ions and the following static modifications: carboxyamidomethylation of cysteine residues (+57.02 Da), dimethylation of lysines (+28.03 Da) and thioacylation of peptide amino termini (+88.00 Da). Semi-style cleavage searches were applied with no constraints for the orientation of the specific terminus. The Web-based PICS service^9^ was used for inference of nonprime sequences and reconstruction of the cleavage sites. Sequence logos were generated with IceLogo^33^, shown are significant differences in amino acid occurrence at each position as compared to the expected amino acid frequency in the human proteome-derived peptide libraries (*p*-value of 5%). The mass spectrometry proteomics data have been deposited to the ProteomeXchange Consortium (http://proteomecentral.proteomexchange.org) via the PRIDE partner repository^34^ with the dataset identifier PXD003078.

### SDS-PAGE and Immunoblotting

Protein samples were analyzed by SDS-PAGE using 12% gels and the Mini Protean III electrophoresis apparatus (Bio-Rad, Hercules, CA, USA). Gels were stained with Coomassie Brilliant Blue R-250 (Sigma). For immunoblotting analysis, protein samples were separated by SDS-PAGE (12% gels) and transferred to a polyvinylidene difluoride membrane (40 V, overnight at 11 °C) in 25 mM Tris and 192 mM Glycine buffer, with 20% methanol using a Trans Blot Electrophoretic Transfer Cell (Bio-Rad, Hercules, CA, USA). The membranes were blocked for 60 min with 5% (w/v) nonfat dry milk plus 0.1% (v/v) Tween-20 in 10 mM Tris pH 8.0 with 150 mM NaCl, and then incubated with the primary antibody for 60 min. Primary antibodies used were mouse anti-His-tag antibody (GenScript; 1:10000 dilution), and rabbit anti-shewasin D antibody (specifically raised towards the sequence Ala-Asn-Met-Thr-Pro-Glu-Ser-Leu-Ala-Leu-Asp-Pro-Leu-Asn, amino acids 208–221) (GenScript; 1:10000 dilution). After several washes with 0.5% (w/v) nonfat dry milk plus 0.1% (v/v) Tween-20 in 10 mM Tris and 150 mM NaCl pH 8.0, the membranes were incubated for 60 min with the secondary antibody: [alkaline phosphatase-conjugated goat anti-(mouse IgG + IgM)(GE Healthcare; 1:10 000 dilution) or [alkaline phosphatase-conjugated goat anti-(rabbit IgG + IgM)(GE Healthcare; 1:10 000 dilution). The membranes were again washed and alkaline phosphatase activity was visualized using ECF (GE Healthcare) on a Molecular Imager FX (Bio-Rad).

## Additional Information

**How to cite this article**: Leal, A. R. *et al*. Enzymatic properties, evidence for *in vivo* expression, and intracellular localization of shewasin D, the pepsin homolog from *Shewanella denitrificans. Sci. Rep.* **6**, 23869; doi: 10.1038/srep23869 (2016).

## Supplementary Material

Supplementary Information
