# Supplementary material for: Enzymatic properties, evidence for in vivo expression, and intracellular localization of shewasin D, the pepsin homolog from Shewanella denitrificans
Source: Sci Rep. 2016 Mar 31;6:23869. doi: 10.1038/srep23869 (PMC4814920; doi:10.1038/srep23869)
Supplement: Supplementary Information [file srep23869-s1.pdf]

Enzymatic properties, evidence for *in vivo* expression, and intracellular localization of shewasin D, the pepsin homolog from *Shewanella denitrificans*

Ana Rita Leal[1,2§], Rui Cruz[1,2], Daniel Bur[3], Pitter F. Huesgen[4], Rosário Faro[1], Bruno Manadas[1,2], Alexander Wlodawer[5], Carlos Faro[1,2], Isaura Simões[1,2*]

[1]CNC-Center for Neuroscience and Cell Biology, University of Coimbra, 3004-517 Coimbra, Portugal; [2]Biocant, Biotechnology Innovation Center, 3060-197 Cantanhede, Portugal; [3]Actelion Pharmaceuticals Ltd, CH-4123 Allschwil, Switzerland; [4]Central Institute for Engineering, Electronics and Analytics, ZEA-3, Forschungszentrum Jülich, 52425 Jülich, Germany; [5]Protein Structure Section, Macromolecular Crystallography Laboratory, National Cancer Institute at Frederick, Frederick, MD 21702 Maryland, USA;

SUPPLEMENTARY DATA

MATERIALS AND METHODS

Pepstatin-agarose affinity chromatography. A *S. denitrificans* culture was grown in a final volume of 2 L of Marine broth pH 7.6 at 30 °C for 16 h. The culture was then harvested by centrifugation, resuspended in 0.05 M sodium acetate buffer pH 4.0 containing 0.2 M NaCl, 5 mM EDTA, 0.01 mM E-64 and 1 mM Pefabloc and the cell suspension lysed with three passages through an EmulsiFlex (AVESTIN, Inc.) (10000 psi). The soluble fraction separated by centrifugation (186000x*g*, for 20 min) was then incubated for 45 minutes with 0.5 mL pepstatin-agarose resin (Sigma-Aldrich), pre-equilibrated with 0.05 M sodium acetate buffer pH 4.0 buffer containing 0.2 M NaCl. Washes were performed with 0.05 M sodium acetate buffer pH 4.0 with 0.2 M NaCl and protein elution was carried out with 0.05 M Tris-HCl buffer pH 9.0.

FIGURES

FIGURE S1

```
atgagcaaacattttattccgctgccgctgaccaacgtgctggcggatggtggatatagc
 M  S  K  H  F  I  P  L  P  L  T  N  V  L  A  D  G  G  Y  S 
gcgagcgtgtgcctgggcagccagctggcgaaagtgaacctgattattgataccggcagc
 A  S  V  C  L  G  S  Q  L  A  K  V  N  L  I  I  D  T  G  S 
agcaccctggtggtgcatgaaaaccgttatcagggcatgcatgatacccgtctgcagagc
 S  T  L  V  V  H  E  N  R  Y  Q  G  M  H  D  T  R  L  Q  S 
accagcctggcgcagcaagtgagctatggcgtgggcggctggtttggcagcgtggtgcat
 T  S  L  A  Q  Q  V  S  Y  G  V  G  G  W  F  G  S  V  V  H 
acccgttttaacattctggatgtgagcgtggatgatatgccgctggcgctggtgcatcat
 T  R  F  N  I  L  D  V  S  V  D  D  M  P  L  A  L  V  H  H 
gaagcggaacatagctttattaacgcggatggcatttggggcatggcgtatcatagcctg
 E  A  E  H  S  F  I  N  A  D  G  I  W  G  M  A  Y  H  S  L 
aaccgtagctatgatatgagcgaatatctgaccgcgaacgcgattgcacctccagcgacc
 N  R  S  Y  D  M  S  E  Y  L  T  A  N  A  I  A  P  P  A  T 
tatccgtggccgtttccggaaaacgatcagccgcagtttccgctgccggaatttcagagc
 Y  P  W  P  F  P  E  N  D  Q  P  Q  F  P  L  P  E  F  Q  S 
gaagcgagcgattttaaacagctgattcatggcctgccggaacaggatgtggcgaccgcg
 E  A  S  D  F  K  Q  L  I  H  G  L  P  E  Q  D  V  A  T  A 
tttaccctgatggaacagcagggcctggttagcaaccgttttgcgtttattgcgcatcgt
 F  T  L  M  E  Q  Q  G  L  V  S  N  R  F  A  F  I  A  H  R 
agcagcattcatcatgcgaaagcgaacatgaccccggaaagcctggcgctggatccgctg
 S  S  I  H  H  A  K  A  N  M  T  P  E  S  L  A  L  D  P  L 
aaccagggcatgctgattctgggcggcgatgaacgtctgagccagtattttcagggcgaa
 N  Q  G  M  L  I  L  G  G  D  E  R  L  S  Q  Y  F  Q  G  E 
tttaccgatctgaaagtggtgcatgatcgttattataacgtgaacctgctgagcctgagc
 F  T  D  L  K  V  V  H  D  R  Y  Y  N  V  N  L  L  S  L  S 
ctggctggtggagcaccgattaaactgccgagcgcgctggaagcgcagctgagccgtggc
 L  A  G  G  A  P  I  K  L  P  S  A  L  E  A  Q  L  S  R  G 
agcagcaacgcgattattgataccggcgcgagcctggttagcctgccgagccaggcgttt
 S  S  N  A  I  I  D  T  G  A  S  L  V  S  L  P  S  Q  A  F 
acccaagtgattgcggaactgagccagaccgtgccgcaggcggcgaaactgctggcgccg
 T  Q  V  I  A  E  L  S  Q  T  V  P  Q  A  A  K  L  L  A  P 
tttattggcgatattaacaaagtgacccaggcgcagagccagggcattgcgatgagcgaa
 F  I  G  D  I  N  K  V  T  Q  A  Q  S  Q  G  I  A  M  S  E 
ctgaacctggcgctgtggccggcgctggaatttcattttgaaggcgcggcggatggccgt
 L  N  L  A  L  W  P  A  L  E  F  H  F  E  G  A  A  D  G  R 
gcgagcctgagctgcccggccgaatgctattggcagctgaacagcccggcccccgggtcgt
 A  S  L  S  C  P  A  E  C  Y  W  Q  L  N  S  P  A  P  G  R 
gccgtgtttaaactgatgggtcagctggccggttggccggcccagagcattctgggtctg
 A  V  F  K  L  M  G  Q  L  A  G  W  P  A  Q  S  I  L  G  L 
ccgctgctgaacccgtatttttgtgctgtttcagcgtgatagcggcgaatttggcaccgtg
 P  L  L  N  P  Y  F  V  L  F  Q  R  D  S  G  E  F  G  T  V 
cgttttgcggcgcatcgtcagaaaagctga
 R  F  A  A  H  R  Q  K  S  - 
```

Figure S1: Nucleotide sequence of codon-optimized shewasin D gene and the deduced amino acid sequence.

FIGURE S2

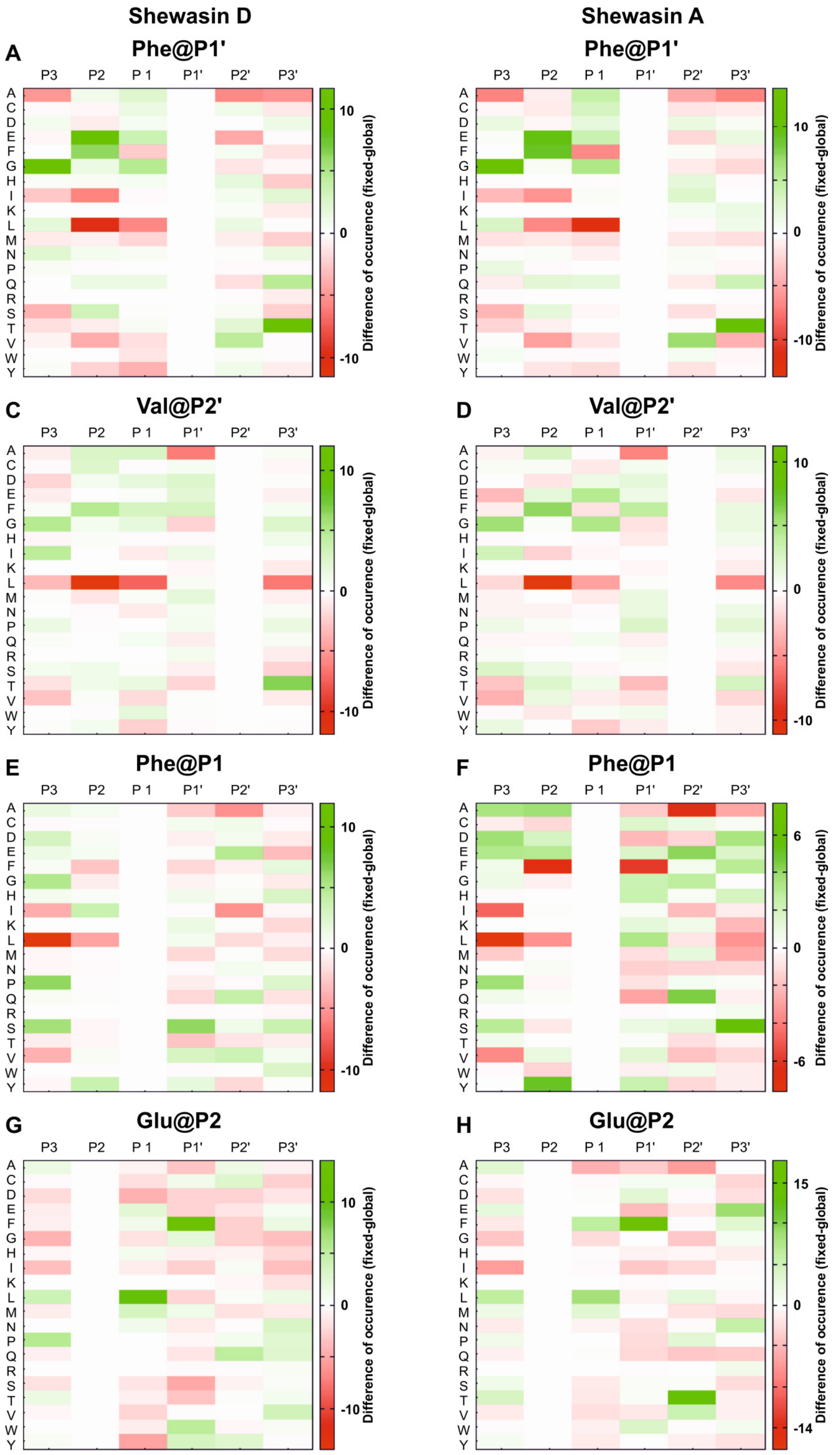
Shewasin D
Shewasin A
A
Phe@P1'
B
C
Val@P2'
D
E
Phe@P1
F
G
Glu@P2
H
P3 P2 P 1 P1' P2' P3'
A C D E F G H I K L M N P Q R S T V W Y
Difference of occurence (fixed-global)
10
0
-10
6
-6
15
-14

Figure S2: Subsite cooperativity analysis for shewasin D and shewasin A. Using web-based PICS analysis (1), different amino acid residues were fixed at selected positions in order to visualize the amino acids that stay in positive (green) or negative (red) cooperative relation with each fixed residue. Subsite cooperativity for shewasin D and shewasin A was analyzed by fixing Phe at P1' (A and B), Val at P2' (C and D), Phe at P1 (E and F), and Glu at P2 (G and H) positions. (A) Of the 126 unique cleavage sites containing Phe in P1', 37 had Leu in P1, and 21 had Glu in P2. (B) Of the 107 unique cleavage sites that contained Phe in P1', 30 had Leu in P1, and 15 had Glu in P2. (C) Of the 177 unique cleavage sites that contained Val in P2', 49 had Leu in P1. (D) Of the 176 unique cleavage sites that contained Val in P2', 63 had Leu in P1. (E) Of the 162 unique cleavage sites that contained Phe in P1, 12 had Phe in P1'. (F) Of the 117 unique cleavage sites that contained Phe in P1, 3 had Phe in P1'. (G) Of the 90 unique cleavage sites that contained Glu in P2, 21 had Phe in P1'. (H) Of the 57 unique cleavage sites that contained Glu in P2, 15 had Phe in P1'.

FIGURE S3

A

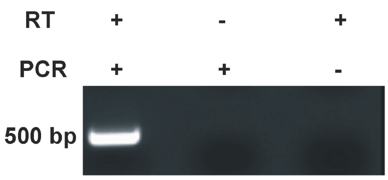

B

```
S     ------------------------CTACCCAGCGCCCTTGAGGCCCAGCTTAGTCGCGGT 36
SD    CTTGCTGGTGGAGCGCCTATCAAGCTACCCAGCGCCCTTGAGGCCCAGCTTAGTCGCGGT 840
                              ************************************

S     TCAAGCAATGCCATCATAGACACGGGCGCCTCCTTAGTGTCTTTGCCCTCCCAAGCCTTC 96
SD    TCAAGCAATGCCATCATAGACACGGGCGCCTCCTTAGTGTCTTTGCCCTCCCAAGCCTTC 900
      ************************************************************

S     ACTCAAGTTATCGCAGAGTTAAGCCAAACTGTGCCACAGGCCGCTAAGTTATTGGCGCCT 156
SD    ACTCAAGTTATCGCAGAGTTAAGCCAAACTGTGCCACAGGCCGCTAAGTTATTGGCGCCT 960
      ************************************************************

S     TTTATTGGTGATATTAACAAGGTCACACAGGCACAGAGTCAAGGTATTGCCATGAGTGAG 216
SD    TTTATTGGTGATATTAACAAGGTCACACAGGCACAGAGTCAAGGTATTGCCATGAGTGAG 1020
      ************************************************************

S     CTTAATTTAGCCCTTTGGCCAGCACTAGAGTTTCACTTTGAAGGCGCCGCGGATGGGCGA 276
SD    CTTAATTTAGCCCTTTGGCCAGCACTAGAGTTTCACTTTGAAGGCGCCGCGGATGGGCGA 1080
      ************************************************************

S     GCAAGCCTTAGTTGCCCTGCCGAATGTTATTGGCAGCTCAATAGCCCAGCCCCAGGTCGC 336
SD    GCAAGCCTTAGTTGCCCTGCCGAATGTTATTGGCAGCTCAATAGCCCAGCCCCAGGTCGC 1140
      ************************************************************

S     GCCGTGTTTAAACTCATGGGTCAACTGGCAGGCTGGCCGGCCCAGAGCATACTCGGGCTG 396
SD    GCCGTGTTTAAACTCATGGGTCAACTGGCAGGCTGGCCGGCCCAGAGCATACTCGGACTG 1200
      ******************************************************** ***

S     CCGCTACTCAACCCATACTTTGTGTTGTTTCAGCGCGATAGCGGCGAATTTGGCACAGTG 456
SD    CCGCTACTCAACCCATACTTTGTGTTGTTTCAGCGCGATAGCGGCGAATTTGGCACAGTG 1260
      ************************************************************

S     CGCTTTGCCGCCCATAGGCAAAAATCATGA 486
SD    CGCTTTGCCGCCCATAGGCAAAAATCATGA 1290
      ******************************
```

Figure S3: Shewasin D gene is transcribed in *Shewanella denitrificans*. (A) RT-PCR analysis of Sden_0804/shewasin D expression (RT +/ PCR +); negative control for the cDNA synthesis lacking reverse transcriptase (RT - / PCR +); negative control of the PCR reaction (RT + / PCR -). An amplification product of 496 bp was expected. (B) Nucleotide

sequence alignment of the cDNA product amplified by RT-PCR (S) with shewasin D gene sequence (SD) (GeneID: Sden_0804). Underlined regions correspond to primer sequences used for RT-PCR analysis.

FIGURE S4

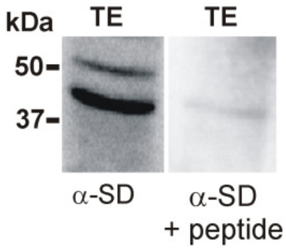

Figure S4: Specificity of anti-shewasin D antibody using peptide competition assays. Total extract (TE) of *S. denitrificans* cells were analyzed by Western blot with anti-shewasin D antibody (left panel). As a control for non-specific staining, peptide competition assays were performed by blocking the anti-shewasin D antibody with 100-fold (mass) excess of immunizing peptide (ANMTPESLALDPLN) for 20 minutes at room temperature prior immunoblotting analysis (right panel).

FIGURE S5

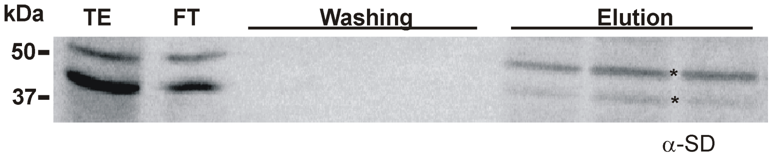

Figure S5. Interaction of native shewasin D with pepstatin-agarose. Western blot analysis of native shewasin D purification by pepstatin-agarose affinity chromatography. Total soluble extract (TE) of *S. denitrificans* cells resuspended in 0.05 M sodium acetate pH 4.0 buffer containing 0.2 M NaCl, 5 mM EDTA, 0.01 mM E-64 and 1 mM pefabloc was incubated for 45 minutes with pepstatin-agarose resin, pre-equilibrated with 0.05 M sodium acetate buffer pH 4.0, 0.2 M NaCl. Resin was then washed with 0.05 M sodium acetate buffer pH 4.0, 0.2 M NaCl. Elution was carried out with 0.05 M Tris-HCl buffer pH 9.0. Total soluble extract (TE), flow-through (FT), washing and eluted samples were analyzed by Western blot with anti-shewasin D antibody. Both forms immunodetected by the antibody are highlighted with an asterisk.
